# Efficacy of transcranial direct current stimulation on seizure control in patients with refractory epilepsy: a systematic review and meta-analysis of randomized controlled trials

**DOI:** 10.1007/s10143-025-03657-0

**Published:** 2025-06-19

**Authors:** Nada Ibrahim Hendi, Yaser AbuSammour, Mohamed Khaled, Ahmed S. Mohamed, Ahmed Mostafa Amin, Mohamed Saleh Fallaha, Basma Kamel, Yehia Nabil Abdalla Helmy, Mohamed Ali Saeed Hassan, Mostafa Meshref

**Affiliations:** 1 Faculty of Medicine, Ain Shams University, Cairo, Egypt; 2 Faculty of Medicine, Al-Balqa Applied University, Al-salt, Jordan; 3 Faculty of Medicine, Alexandria University, Alexandria, Egypt; 4 Faculty of Medicine, Merit University, Sohag, Egypt; 5 Faculty of Medicine, AL-Azhar University, Cairo, Egypt; 6 Faculty of Medicine, Mansoura University, Daqahleya, Egypt; 7 Faculty of Medicine, Zagazig University, Sharkeya, Egypt; 8 Department of Neurology, Faculty of Medicine, Al-Azhar University, Cairo, Egypt; 9 Supervisor of Epilepsy Units, Faculty of Medicine, Al-Azhar University Hospitals, Al-Azhar University, Cairo, Egypt

**Keywords:** Drug-resistant epilepsy, Non-invasive neurostimulation, Seizure frequency, Transcranial direct current stimulation, tDCS

## Abstract

Drug-resistant epilepsy is a challenging condition that affects around 30% of all patients with epilepsy. Evidence regarding treatment options is limited, especially for surgery and invasive techniques. However, non-invasive techniques constitute a promising alternative for these patients. This meta-analysis aims to evaluate the effectiveness of transcranial direct current stimulation on seizure frequency management in patients with drug-resistant epilepsy. We searched the literature in PubMed, Scopus, and Web of Science up to December 2023. We included randomized controlled trials that compared transcranial direct current stimulation with sham stimulation. Our main outcomes of interest were a percentage reduction in seizure frequency and epileptiform discharge frequency. A total of 10 studies with 269 patients were included. Monthly seizure frequency was significantly reduced by an average of -45.39%and -39.34% at week 4 and week 8, respectively. There was a significant reduction in IED in favor of tDCS at week 2 (SMD = -0.87, 95% CI = [− 1.49, − 0.25], *P* = 0.006), 4 weeks (SMD = -1.17, 95% CI = [− 1.67, − 0.66], *P* < 0.00001, Moderate quality of evidence) and 8 weeks (SMD = -1.11, 95% CI = [− 1.69, − 0.53], *P* = 0.0002) of follow-up. There were no serious adverse events associated with the stimulation. Transcranial direct current stimulation was associated with a reduction in both seizure frequency and epileptiform discharges with minimal side effects. Further studies with larger sample sizes and consensus protocol guidelines are needed to verify its long-term safety and effectiveness.

**Supplementary Information:**

The online version contains supplementary material available at 10.1007/s10143-025-03657-0.

## Clinical trial number

Not applicable.

**Supplementary Information:**

The online version contains supplementary material available at 10.1007/s10143-025-03657-0.

## Key summary points

- This Meta-analysis aims to assess whether non-invasive transcranial direct current stimulation is a safe and effective therapeutic option for seizure frequency reduction in patients with focal drug-resistant epilepsy.
- The pooled results from 10 randomized controlled trials showed a significant seizure frequency reduction in patients with focal drug-resistant epilepsy who received transcranial direct current stimulation group.
- There was a moderate level of certainty evidence regarding the percentage reduction of seizure frequency outcome in patients with focal drug-resistant epilepsy.
- There were no serious adverse events associated with the stimulation in patients with focal drug-resistant epilepsy.
- Our findings suggest that transcranial direct current stimulation is a safe and effective non-invasive alternative for proper seizure control in patients with focal drug-resistant epilepsy.

**Supplementary Information:**

The online version contains supplementary material available at 10.1007/s10143-025-03657-0.

## Introduction

Drug-resistant epilepsy (DRE) represents a major challenge affecting more than 30% of all patients with epilepsy [1, 2]. Surgical management of DRE proved to be effective in both children and adults. However, its efficacy is limited in cases of diffuse, multifocal, or inaccessible epileptogenic foci. Moreover, the high failure rate of surgery mandates the shift to other treatment options [2, 3]. Moreover, invasive stimulation techniques such as vagus nerve stimulation (VNS), anterior thalamic deep brain stimulation (DBS), and responsive neurostimulation (RNS) possessed several adverse events such as infection, hematoma at the location of the implant, as well as hoarseness, coughing, and paresthesia [4]. Those adverse events besides the invasive nature of the techniques mandate the shift to non-invasive techniques due to their simplicity, safety, tolerability, and reversibility [5]. Transcranial direct current stimulation (tDCS) is one example with higher safety and lower cost compared to other non-invasive neuro-stimulation techniques such as repetitive transcranial magnetic stimulation [4].

The tDCS involves the application of an electric current of low intensity to the scalp causing subthreshold modulation of the membrane potential leading to depolarization (anodal tDCS) or hyperpolarization (cathodal tDCS) depending on the direction of stimulation [6]. Multiple preclinical trials have shown that tDCS can modulate cortical excitability and significantly raise the seizure threshold in epileptic rodent models [7–9] which paved the way for the first clinical trial in 2006 by Fregni et al. [10] on 19 patients with epilepsy using one session (20 min) of cathodal tDCS that resulted in a significant decrease in seizure frequency. Similarly, a few years later, a significant reduction in epileptiform discharge was observed by Auvichayapat et al. after a single tDCS session on 29 epileptic children [11]. However, the effect of this single-session protocol on seizure reduction was observed only immediately following the session, with no sustained effect over a longer period. Subsequent clinical trials that adopted a multiple tDCS session protocol revealed a more prolonged seizure control [12–14] These discrepancies in results raised the need for a thorough meta-analysis to bring results together and give a better idea of the overall impact.

Some recent reviews have suggested the efficacy of cathodal-tDCS (c-tDCS) in reducing seizure frequency in patients with refractory epilepsy [15, 16]. However, these results included patient-related, device-related, and protocol-related heterogeneities in methodology that may impact the validity and generalizability of the findings. Therefore, we conducted this meta-analysis to evaluate the direct effect of cathodal-tDCS on patients with refractory epilepsy while investigating the potential impact of methodological differences (e.g. variations in devices utilized, different current intensities and durations and multiple follow up points) on the outcomes reported.

## Method

This systematic review and meta-analysis strictly adhered to the Preferred Reporting Items for Systematic Review and Meta-Analysis (PRISMA) statement guidelines [17]. Our study protocol was prospectively registered in the International Prospective Register of Systematic Reviews (PROSPERO), (registration number: CRD42024496888).

### Literature search strategy

We searched for included studies in PubMed, Scopus, and Web of Science from inception till Dec 18, 2023. No restrictions or filters were applied. A detailed description of our search strategy and keywords is attached to the supplementary file. (Table S1 in the supplementary file)

### Eligibility criteria and study selection

We screened the obtained records for inclusion using Rayyan software [18]. Screening was done by two independent authors in two steps: title and abstract screening, followed by full-text screening. Disagreements were resolved by consensus or by referring to the first author. We included studies with the following criteria: (A) population: Patients with drug-resistant epilepsy defined by the international league against epilepsy (ILAE) as failure of two or more antiepileptic drugs either given alone or in combination [1](B) Intervention: tDCS with no restriction on intensity, duration, or number of stimulation sessions, (C) Comparison: Sham stimulation, (D) Outcome: Seizure frequency or interictal epileptiform discharge, and (E) study design: randomized controlled trials (RCTs). Studies were excluded if they were non-randomized or non-controlled. Moreover, we excluded studies that didn’t have solid criteria and evidence about drug resistance in epilepsy. Observational studies, editorials, letters, book chapters, conference papers, case reports, reviews, single-arm studies, and research published in languages other than English were also excluded from our study.

### Data extraction

Two authors independently extracted data about the study setting and summary, baseline characteristics of the included participants, and the outcomes of interest using a predefined extraction sheet. Conflicts were resolved by consensus or referral to the first author.

### Quality assessment

Two independent authors assessed the quality of the included studies using the Cochrane risk of bias assessment tool for RCTs version two (ROB2) [19]. The overall authors’ judgment for each domain fell into three categories: low, some concerns, or high risk of bias. Conflicts were resolved by consensus or referral to the first author.

The quality of the synthesized evidence for the primary outcomes was assessed using the GRADE (grading of recommendations, assessment, development, and evaluation) (Table S2 in the supplementary file).

### Measures of treatment effect and data synthesis

Our outcomes of interest were percentage reduction of seizure frequency (SF) and interictal epileptiform discharge frequency (IED). Data was obtained as means and standard deviations. Graphical data was obtained using the web plot digitizer software (Plot Digitizer, version 2.6.8, Free Software Foundation, Boston, MA, USA). Outcomes were pooled as standardized mean difference (SMD) with a 95% confidence interval (CI) using the inverse variance method on RevMan 5.4. We used the random effect model due to the significant variability in the assessment methods among included studies.

### Subgroup analysis

Because the included studies varied in the stimulation protocol and follow up duration, we stratified the included studies in different subgroup analyses based on the follow-up point of assessment, intensity of active stimulation, duration of the stimulation session, number of sessions, overall risk of bias, and method of assessment.

### Assessment of heterogeneity

Heterogeneity was evaluated by visual assessment of the forest plots. The qualitative assessment of heterogeneity was assessed by a P value of less than 0.1 for the chi-square test. Whereas, quantification of the magnitude of heterogeneity is assessed by the *I*-square test. In case of significant heterogeneity, we performed a sensitivity analysis (leave one out analysis) to assess the effect of single study removal on the overall effect size.

### Assessment of publication bias

Due to the small number of included trials (less than 10), publication bias was assessed by STATA software using the DOI plot and Luis Furuya-Kanamori index (LFK index) which showed higher sensitivity than Egger’s test and the funnel plot in the case of a small number of studies. DOI plot is a graphical method to visualize the asymmetry of study effects through a normal quantile versus effect plot. LFK index detects and assesses the asymmetry of study effects quantitatively based on the DOI plot. LFK index values of ≤ ± 1, >±1 but < ± 2, and ≥ ± 2 were considered no, minor, and major asymmetry respectively [20].

## Results

### Description of included studies

**Fig. 1:**
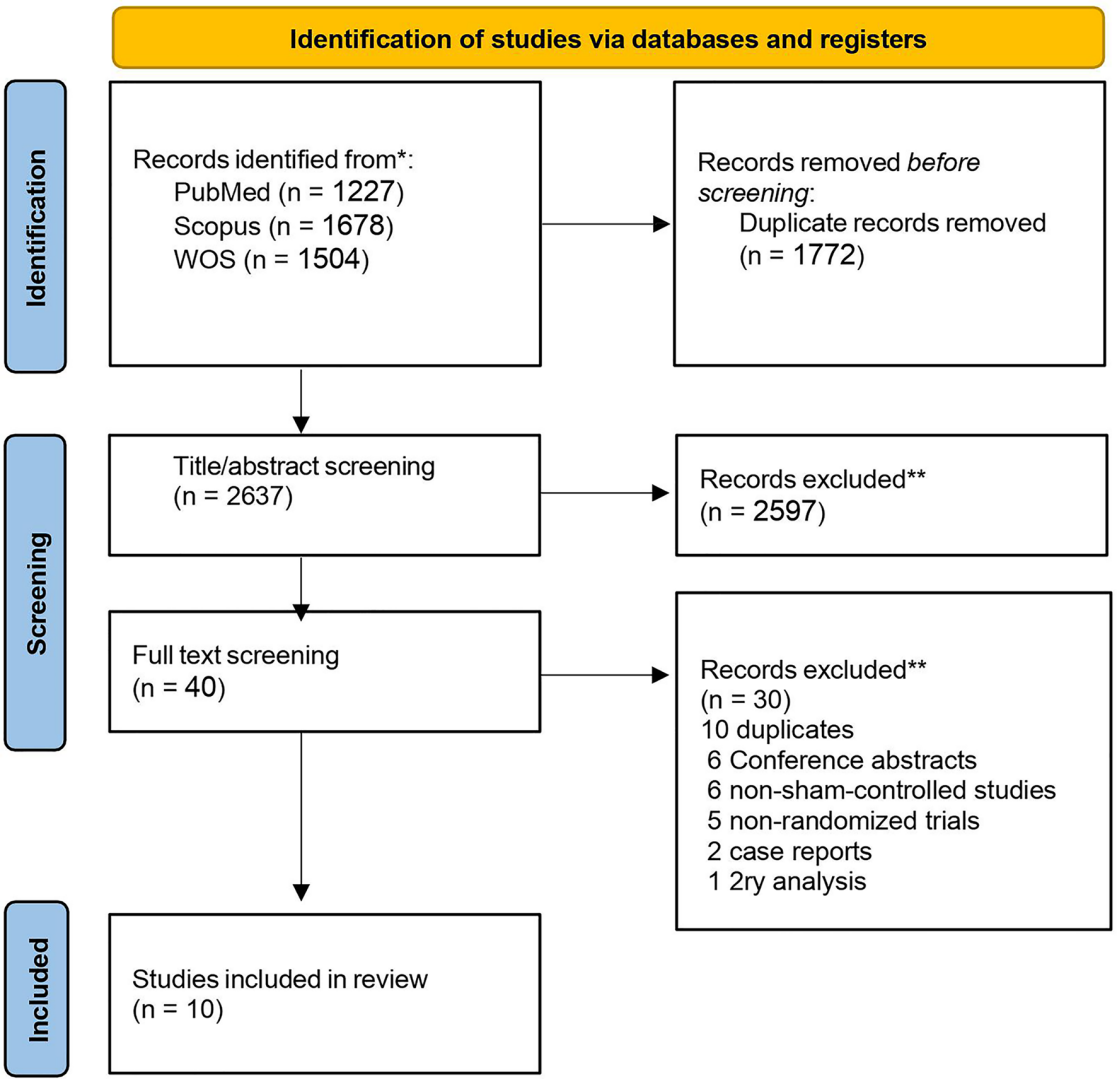
PRISMA flowchart

Our database search retrieved 4406 articles. 1772 duplicates were removed and 2597 records were excluded in the title and abstract screening. The remaining studies underwent full-text screening, after which we included 10 RCTs in our systematic review and eight of them were eligible for meta-analysis. An updated search on Dec 18 2023 was done which didn’t retrieve any additional studies. Two of the included studies had two intervention groups compared to a placebo group. We added them to the analysis as two separate studies and referred to them as studies A and B. A detailed description of the selection process is described in the PRISMA flow diagram (Fig. 1).

We included a total of 10 RCTs comprising 264 patients who had DRE. Of the 10 RCTs, two studies were on children [11, 13]one study was on adolescents [21]and the rest were on adult patients. All of them used the International System of Electrode Positioning 10–20 except Rezakhani et al. [22] used the 10–10 system. Almost all included studies reported seizure frequency and epileptiform discharge as the primary outcomes. The summary of the included studies and the baseline characteristics of their included patients are shown in (Table 1), and (Table S3 in the supplementary file) respectively.

**Table 1:** Summary of the included studies

First Author, Year, design	Country	Epilepsy type, Etiology	Sample size, Age group	Cathode position	Anode Position	Contact area of the electrode	Current intensity	Sessions number and duration	Follow-up duration	Safety findings	Main findings
**Fregni 2006** (Parallel)	Brazil	Focal, MCDs	19 Young adults	EEG 10–20 focus	silent area or contralateral head	35 cm^2^	1 mA	1 session (20 min)	30 days	Itching	1) significant reduction in the number of epileptiform discharges (64.3%) 2) 44% SF decrease in the active group
**auvichayapat 2013** (Parallel)	Thailand	Focal, idiopathic	36 Children	EEG 10–20 focus	contralateral shoulder	35 cm^2^	1 mA	1 session (20 min)	30 days	Transient skin erythema (1 patient)	1) 4.8% SF decrease in the active group for 4 weeks follow-up 2) 57.6% IED reduction in the active up to 48 h.
**auvichayapat 2016** (Parallel)	Thailand	Combined (Lennox-Gastaut Syndrome)	22 Children	Left primary motor cortex (M1)	contralateral shoulder	35 cm^2^	2 mA	5 Sessions (30 min)	4 weeks	Superficial skin burn (1 patient)	1) 55.96% SF decrease in active tDCS at 4 weeks follow-up 2) IED reduction in the active group 42.66% and 8.56% at 2- and 4-week follow-up respectively
**Tekturk 2016** (Crossover)	Turkey	Focal, hippocampal sclerosis (MTLEHS)	12 Adults	temporal region, either T3 or T4	contralateral supraorbital	35 cm^2^	2 mA	3 sessions (30 min)	2 months		84% SF decrease following active tDCS, up to 1 month
**Zoghi 2016** (Parallel)	Australia	Focal (Temporal lobe epilepsy), Variable etiologies	29 Adults	temporal lobe	contralateral supraorbital		1 mA	1 session 18 min (9 + 9)	4 weeks		1) The experimental group showed a significant increase in SICI 2) 42% SF decrease in the active group
**Assenza 2017** (crossover)	Italy	Focal (Temporal lobe epilepsy)	10 Adults	EEG 10–20 focus	contralateral homologous region.	35 cm^2^	1 mA	1 session (20 min)	45 days	Itching	71% SF decrease at 1-week follow-up
**San-Juan 2016** (Parallel)	Mexico.	Focal, hippocampal sclerosis (MTLEHS)	28 Adults	EEG 10–20 focus	silent supraorbital area	35 cm^2^	2 mA	3 or 5 (30 min)	2 months	Mild itching Moderate headache	1) The mean reduction of SF at two months in both active groups was significantly higher than placebo (-48%, -43 vs. -6.25%) 2) A significant IED reduction in all groups immediately after treatment but not at further follow-up points.
**Yang 2020** (Parallel)	China	Focal, Varied	70 Adults	EEG 10–20 focus	contralateral, silent area		2 mA	14 or 2*14 (20 min)	56 days	N/A	50.73 − 21.91% (1x/ day) and 63.19 − 49.79 (2x/day) SF decrease, up to 4–5 weeks
**Rezakhani 2022** (Parallel)	Iran	Focal	20 Adults	HD-tDCS using EEG 10–10 system	F7, FP1, FP2		2 mA	10 sessions (30 min)	3 months		1) SF significantly decreased at 1- and 2- months follow-up 2) EEGs showed a considerable decrease in the frequency of IED at 1-,2- and 3 months follow-up 3) two groups showed no significant difference in their MoCA scores in the first month of follow-up 4) overall QoLIE-89 is significantly higher in the Active group
**Ashrafzadeh 2023** (Parallel)	Iran.	Focal, unknown	18 Adolescents	EEG 10–20 focus	contralateral deltoid muscle		1 mA then 1.5 mA then 2 mA	5 sessions (20 min)	1 month		No significant change in SF nor IED between the 2 groups

EA, Epileptiform activity; EEG, Electroencephalography; HD-tDCS, High-definition tDCS; IED, Interictal Epileptiform discharges; MCD, Malformation of cortical development; MoCA, Montreal Cognitive Assessment; MTLEHS, Mesial temporal lobe epilepsy with hippocampal sclerosis; QoLIE-89, Quality of Life in Epilepsy Inventory; SF, Seizure frequency; SICI, Short interval intracortical inhibition; tDCS, Transcranial direct current stimulation

### Quality assessment

**Fig. 2:**
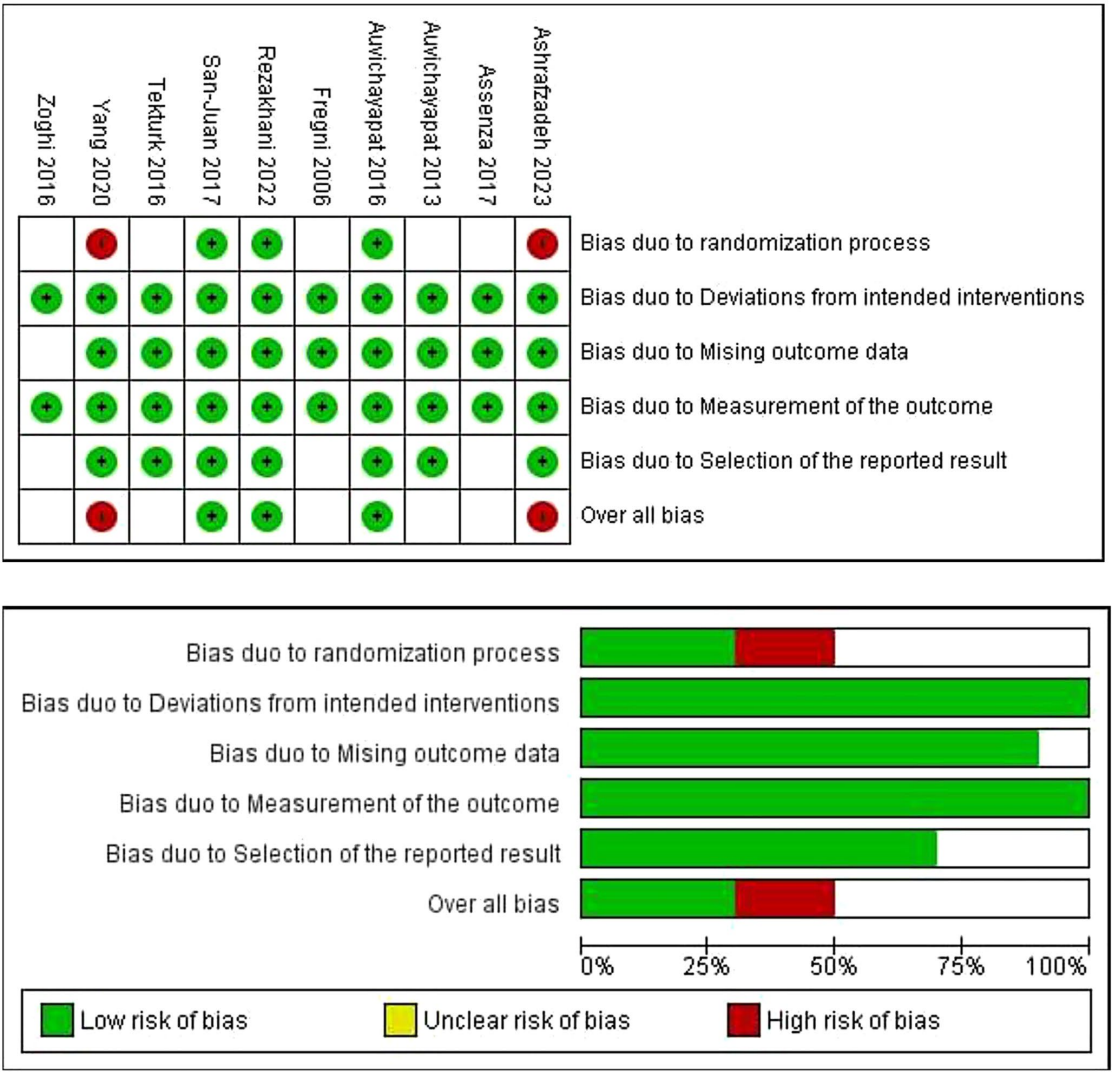
The risk of bias summary and graph according to the cochrane risk of bias assessment tool 2

According to ROB-2, two trials had a high risk of bias, five had a moderate risk, and three had a low risk of bias. The risk of bias was mainly due to issues in the randomization and selection of the reported results domains. The risk of bias graph and summary are shown in (Fig. 2).

### Seizure frequency (SF)

#### Time of seizure frequency assessment in the follow-up

**Fig. 3:**
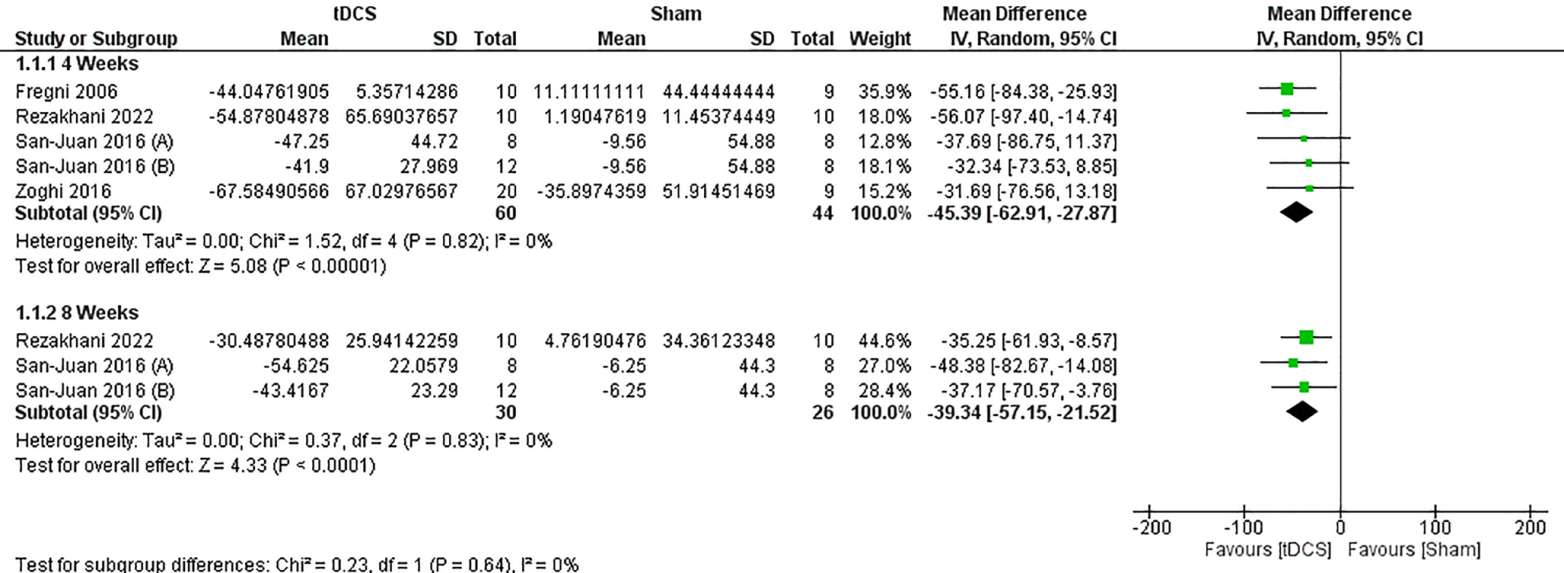
Forest plot of monthly seizure frequency percentage reduction at 4 and 8 weeks

There was a statistically significant reduction of monthly SF at 4 weeks (MD = -45.39, 95% CI = [-62.91, -27.87], *P* < 0.00001), and 8 weeks (MD = -39.34, 95% CI = [-57.15, -21.52], *P* < 0.0001). (Fig. 3).

**Fig. 4:**
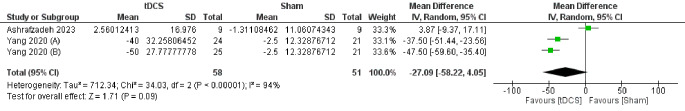
Forest plot of weekly seizure frequency percentage reduction at 4 weeks

However, weekly SF reduction, as reported in 3 trials, showed no significant difference (MD = -27.09, 95% CI = [-58.22, 4.05], *P* < 0.0001). (Fig. 4)

Further detailed subgroup analysis based on the time of assessment is presented in (Fig. S1 in the supplementary file).

#### Intensity of the stimulation

**Fig. 5:**
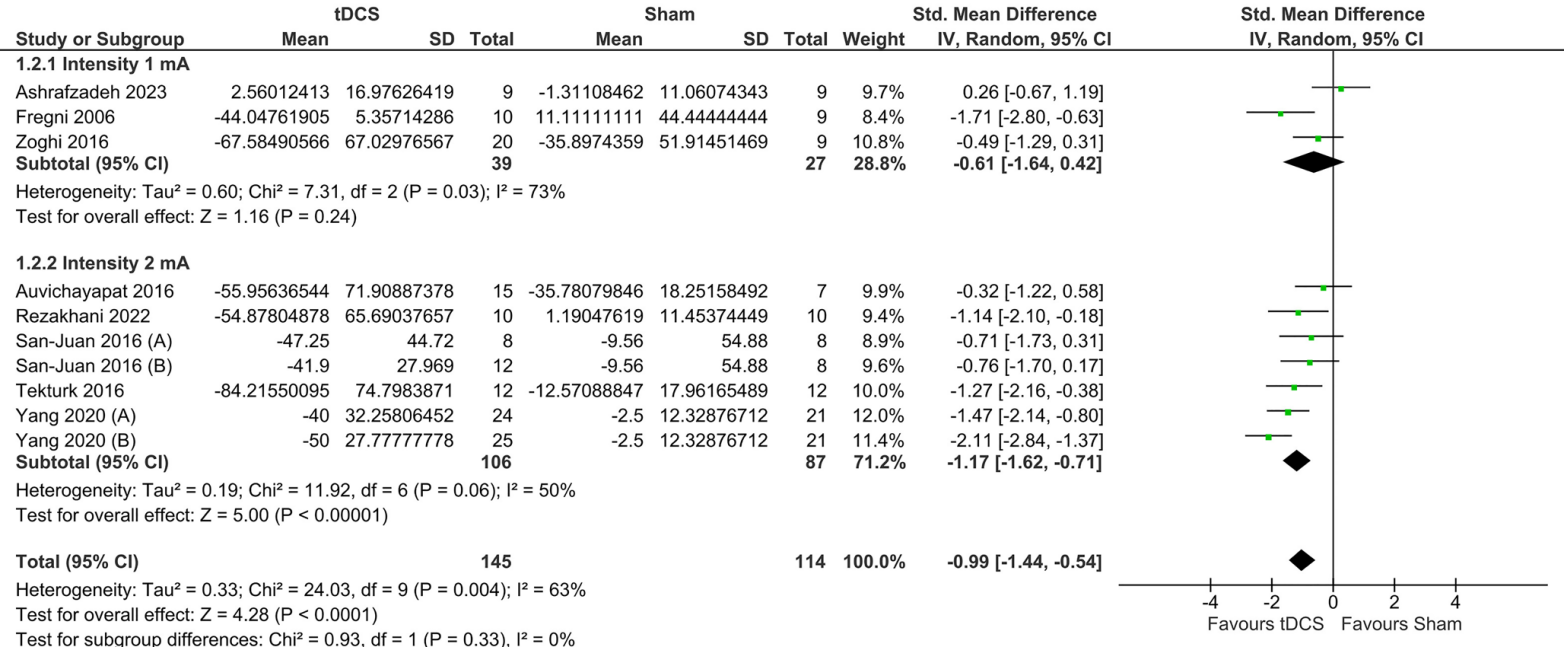
Forest plot of seizure frequency sub-grouped based on the intensity of stimulation

There was a statistically significant difference between tDCS and sham in the 2-mA subgroup (SMD = -1.17, 95% CI = [− 1.62, − 0.71], *P* < 0.00001). However, the test for subgroup differences based on the intensity of stimulation was not significant (*I*^2^ = 0%, *P* = 0.33), which means that current intensity at 1 versus 2 mA does not modify the effect of tDCS. There was significant heterogeneity observed in both subgroups (Fig. 5).

#### Duration of stimulation

**Fig. 6:**
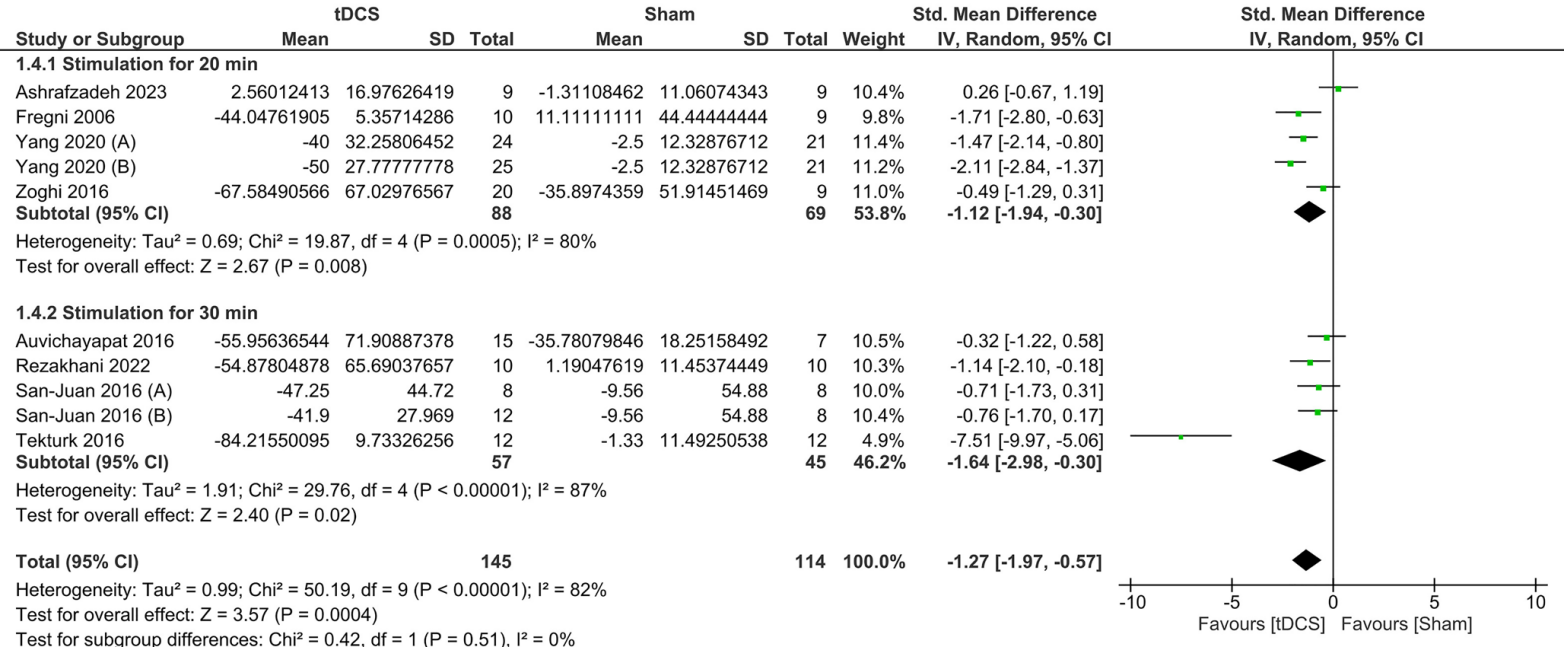
Forest plot of seizure frequency sub-grouped based on the duration of stimulation

There was a significant difference in SF between tDCS and sham in both the 20 min stimulation subgroup (SMD = -1.12, 95% CI = [− 1.94, − 0.30], *P* = 0.008) and the 30 min stimulation subgroup (SMD = -1.64, 95% CI = [− 2.98, − 0.30], *P* = 0.02). There was no significant subgroup difference (*I*^*2*^ = 0%, *P* = 0.51), indicating that there was no overall difference in the effect of tDCS for 20–30 min on seizure frequency in these studies. There was a significant and severe heterogeneity observed among both the 20-min duration subgroup studies [*I*^*2*^ = 80%, *P* = 0.0005], and the 30-min duration subgroup studies [*I*^*2*^ = 87%, *P* < 0.00001] (Fig. 6).

#### Number of stimulation sessions

There is no statistically significant subgroup difference between 1 session versus more than 1 session groups, indicating that the number of stimulation sessions does not modify the effect of tDCS. However, a smaller number of trials and participants contributed data to the case of 1 stimulation subgroup than to the > 1 stimulations subgroup, rendering the analysis may not be able to detect subgroup differences. There was significant heterogeneity in both subgroups. (Fig. S2 in the supplementary file)

### Interictal epileptiform discharge (IED)

#### Time of epileptiform discharge assessment in the follow-up

**Fig. 7:**
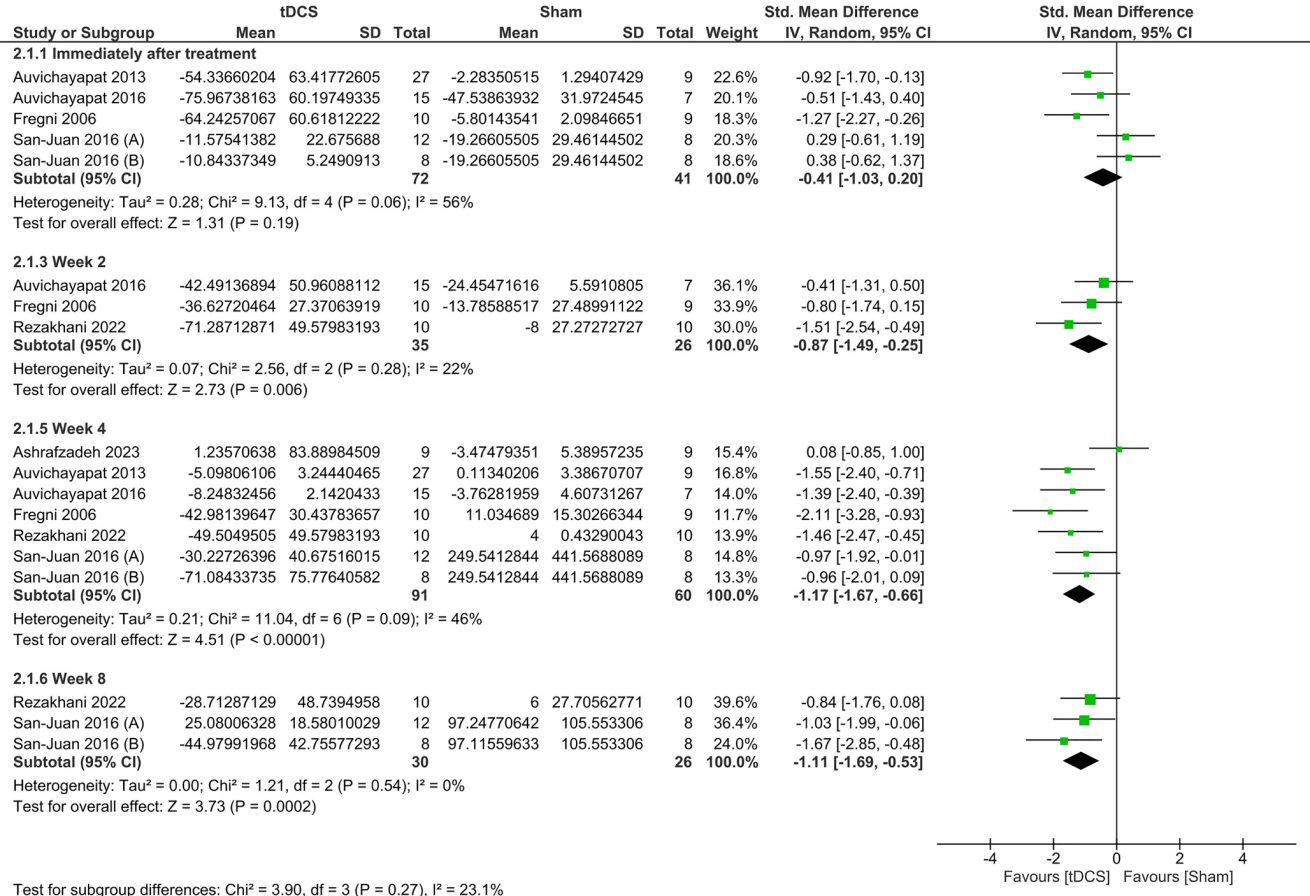
Forest plot of epileptiform discharge at different time points

There was a significant reduction in IED in favor of tDCS at week 2 (SMD = -0.87, 95% CI = [− 1.49, − 0.25], *P* = 0.006), 4 weeks (SMD = -1.17, 95% CI = [− 1.67, − 0.66], *P* < 0.00001, Moderate quality of evidence) and 8 weeks (SMD = -1.11, 95% CI = [− 1.69, − 0.53], *P* = 0.0002) of follow-up. However, there was no significant difference between both groups immediately after treatment (SMD = -0.41, 95% CI = [− 1.03, 0.20]; *P* = 0.19). There was a significant heterogeneity observed in the immediately after treatment [*I*^2^ = 56%, *P* = 0.06], and week 4 subgroups [*I*^2^ = 46%, *P* = 0.09]. (Fig. 7)

#### Intensity of the stimulation

There was no significant subgroup difference between 1 mA and 2 mA subgroups, indicating that current intensity at 1 versus 2 mA does not modify the effect of tDCS on IED. There was significant heterogeneity observed in both subgroups. (Fig. S3 in the supplementary file)

#### Duration of stimulation

There was no significant subgroup difference between the 20 min and 30 min subgroups (*I*^2^ = 0%, *P* = 0.97), indicating that the stimulation duration does not modify the effect of tDCS. No heterogeneity was observed among the 30-min subgroup studies, while the 20-min duration subgroup studies showed substantial heterogeneity. (Fig. S4 in the supplementary file)

#### Number of stimulation sessions

There was no significant subgroup difference between the 1 session and > 1 session subgroups (*I*^2^ = 0%, *P* = 0.07), indicating that the number of stimulation sessions does not modify the effect of tDCS. No significant heterogeneity was observed in any of the subgroups. (Fig. S5 in the supplementary file)

### Sensitivity analysis

Leave-one-out analysis demonstrated the robustness of our results due to the significant differences being not driven by any single study. Heterogeneity in most of the plots was resolved by either removing Auvichaypat 2016 or the high risk of bias studies except for the subgroup 1 mA at which heterogeneity was attributed to the study Fregni et al. and the subgroup of 30 min stimulation at which heterogeneity was attributed to Tekturk et al. Further details on our sensitivity analysis can be found in (Table S4 in the supplementary file).

### Meta-regression

Seizure frequency was positively correlated to the number of stimulation sessions (*P* = 0.026). No significant correlation was found between age and SF or IED. (Fig. S6 & S7 in the supplementary file)

### Cumulative studies analysis

The cumulative analysis of 8 studies showed an overall association between tDCS and SF reduction. A statistically significant association (*P* < 0.05) was achieved from the first study onwards, except when adding Auvichaypat 2016, it brought non-significant results. That explains the contribution of this study to the observed heterogeneity (*I*^*2*^ = 63.23%, *P =* 0.004). (Fig. S8 in the supplementary file)

Cumulative meta-analysis of IED showed statistical significance from the first study onwards. (Fig. S9 in the supplementary file)

### Publication Bias

**Fig. 8:**
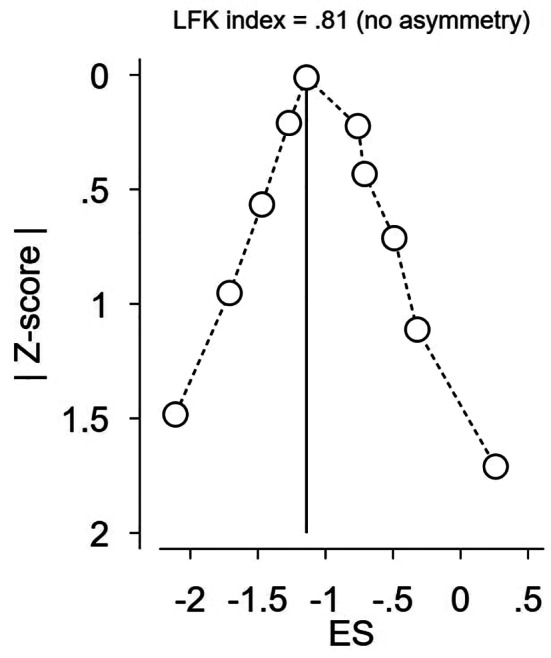
Publication bias assessment (DOI graph) of the seizure frequency outcome at 4 weeks

As shown in the DOI graph (Fig. 8), no asymmetry was detected for either seizure frequency or epileptiform discharge.

**Fig. 9:**
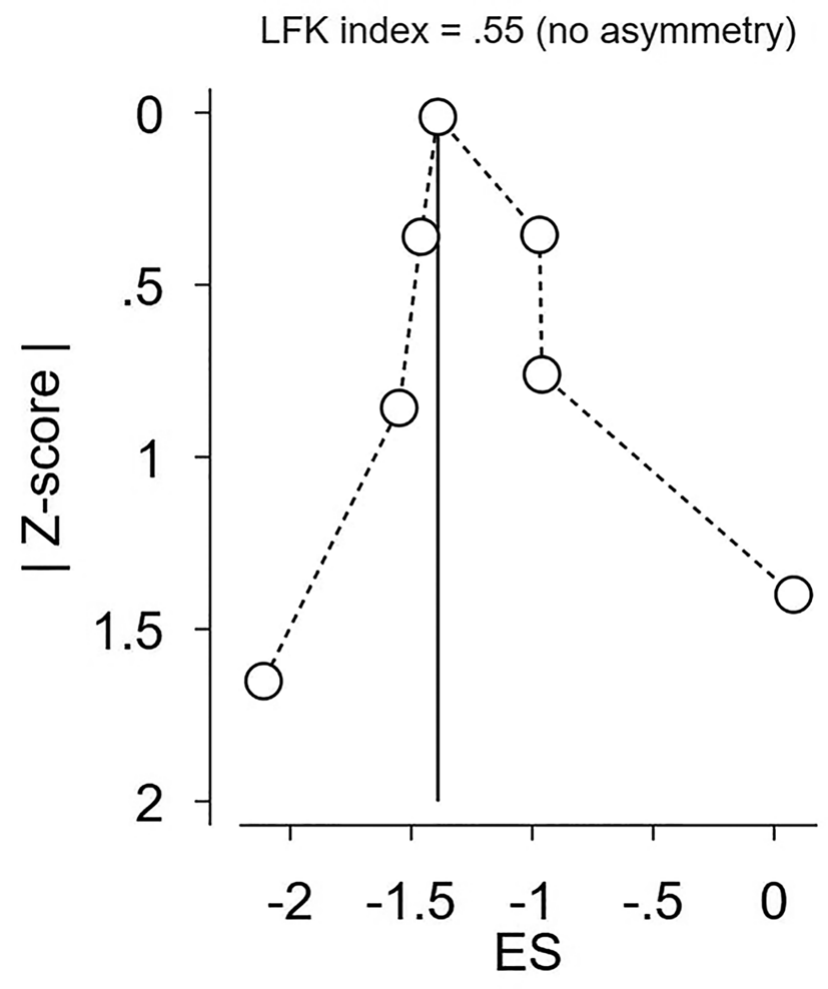
Publication bias assessment (DOI graph) of the epileptiform discharge outcome at 4 weeks

### Safety and tolerability

None of the included trials documented any significant adverse events and tDCS was generally well tolerated among the enrolled patients. The majority had reported mild itching, erythematous rash, or tingling. Additionally, Auvichaypat 2016 reported one case of superficial skin burn, and San Juan reported two cases of headache, one in each group. It’s worth mentioning that all adverse events resolved spontaneously.

## Discussion

### Significance of the study

This meta-analysis was done to assess the efficacy and safety of tDCS in DRE. We found that tDCS leads to a significant reduction in SF compared to sham at all time points from week 1 to week 8 of follow-up. There was no significant subgroup difference between 1 mA and 2 mA or between 20 min stimulation and 30 min stimulation. As regards the IED, it was significantly reduced in the tDCS at all time points except immediately after the treatment subgroup. No difference was found between different intensity or duration subgroups.

### Explanation of our findings

DRE is the failure of two appropriately chosen and well-tolerated antiepileptic drugs either alone or in combination [23]. DRE negatively affects patients’ quality of life, psychological well-being, social interactions, and financial stability [24, 25]. Despite the effectiveness of epileptic surgery, it can be limited in cases of multifocality or inaccessible epileptogenic foci [23]. Additionally, invasive neuro-stimulation approaches showed limited evidence and multiple adverse effects [4, 26].

Our findings on the SF reduction were consistent with previous studies [6, 12, 14, 27, 28]. The tDCS can impact the SF by modifying the membrane potential without causing an action potential. It has a wide range of effects based on the electrode placement and the net effect of the stimulation [4, 16]. Moreover, tDCS can lead to neuronal plasticity in the glutamatergic synapses leading to amplification or prolongation of its effect by what is called long-term potentiation or depression [29, 30]. This effect depends on the stimulation protocol, duration, intensity, and interval [29, 31, 32]. An interval of 20 min was found to be of great efficiency. However, longer intervals may negatively reverse the stimulation effect [29, 30]. Although our study reported similar findings, we failed to demonstrate between group difference which may be attributed to the limited number of studies in each subgroup. Given that epilepsy is a disease of cortical hyperexcitability, it’s justifiable to use tDCS to induce hyperpolarization and thus decrease the activity of the epileptogenic foci [4]. Moreover, it’s also suggested that tDCS can be beneficial for multifocal epilepsy or specific epileptic syndromes such as Lennox-Gastaut syndrome [13]and Rasmussen encephalitis [28, 33]. However, previous studies demonstrated that the outcome can be influenced by different individual variations [34]. Anti-seizure medications (ASM) can influence the reporting of seizure frequency. Kaufmann et al., reported that the use of ASM can lead to underestimation of SF and that it is better to administer Tdcs 48 h after ASM withdrawal. However, no sufficient data was reported in the included studies regarding the state of ASM.

Available literature including the studies of our meta-analysis demonstrated a clinically significant reduction of SF with the use of tDCS which can even reach up to 79% after 4 weeks [6, 11, 14, 35–37]. A previous study reported that using the 20-minute interval approach resulted in a similar SF percentage reduction in the 1 × 20 min and the 2 × 20 min stimulation groups in the 1st month. However, the 2 × 20 min group showed a better long-term effect [6]. The heterogeneity in our analysis of seizure frequency was attributed to the low-quality studies according to ROB. Additionally, we found that Auvichaypat et al. 2016 was an outlier. This could be due to the different sample enrolled being children with Lennox-Gastaut syndrome. On the contrary, in the 1 mA subgroup, heterogeneity was attributed to Fregni et al. This could be explained by the inclusion of patients with exclusive malformation of cortical development, not focal epilepsy [10]. Heterogeneity in the 30 min subgroup was attributed to Tekturk et al., possibly due to the use of sinusoidal alternating current which is thought to be more effective than the classic tDCS leading to the extreme deviation of the results of this study in favor of the stimulation group [37]. 

Generally, our study found a significant SF reduction in favor of the tDCS group. However, some factors could limit the impact and potential significance of this meta-analysis. Most of the included studies used short-term stimulation (< 60 s) in the sham group to give a similar effect to the tDCS. However, there have been some significant concerns raised in the literature that this study protocol does not ensure distinct boundary in the assigned intervention. This might account for the minimal placebo response noted in some of the studies which is greatly lower than what is noted in the literature. For example, some of our included studies reported < 10% placebo effect [6, 10, 21, 22, 28] which contradicts what is observed in almost every large-scale clinical trial in the treatment-resistant epilepsy population, where placebo rates of seizure reduction range from ~ 15–25% [38–41]. On the other hand, a couple of the included trials [37, 42].

There is conflicting data about the effect of tDCS on IEDs. Previous research reported a 64% reduction in IED associated with tDCS on days 0, 15, and 30 of follow-up [10]. Although subsequent studies verified our conclusions [11, 43]other studies demonstrated that there was no notable variation between tDCS and sham in the quantity of spike and sharp waves [21, 44]. Fregni et al. found no significant alteration in the interictal epileptiform discharge but only in the incidence of seizure over a 30-day assessment period [10]. Similar results were also reported by Meiron et al. in a case with early onset epileptic encephalopathy [45]. The patients’ response to tDCS may potentially be affected by individual variances, such as the significant heterogeneity in gyri and sulci patterns among people [34, 46]. Also, Various factors may influence its response, such as anatomical and morphological characteristics, demographic parameters including gender, age, neurochemical factors, and genetic profile. The key elements under investigation are the morphological characteristics, including the thickness of the skull bone, scalp-to-cortical distance, cortex folding, neurotransmitters, and genetic profile [46–48]. This technique is considered a non-invasive, bedside, readily available, and time-efficient technique [49]. However, some tolerable side effects may occur.

A previous meta-analysis conducted by Sun et al., reported a statistically significant SF reduction with tDCS at 4 weeks, but not at 8 weeks They also reported no statistically significant difference in IED [49]. We observed some methodological limitations in this study. Despite the methodological heterogeneity between the studies and variations in the assessment methods, they used fixed-effect model meta-analysis which is not accurate in such cases. For example, the included population, the unit of seizure frequency (per day, week, or month), and the method used for IED assessment differed between studies. In comparison with our meta-analysis, we used a random effect model to overcome such limitations. We also analyzed percentage reduction which gives a more meaningful insight for implementation into clinical practice. We utilized various subgroup analyses to further understand the efficacy and limitations of tDCS. Moreover, we performed GRADE assessment to understand the quality of the obtained evidence.

### Limitations

It’s worth mentioning that our study was not free of limitations. The maximum duration of follow-up was 8 weeks, so it’s unknown whether the positive effects encountered in our study will be sustainable in the long term. Most of our included studies were on the adult population, so we cannot generalize the effect of tDCS on different age groups. There was great variability among the included studies in terms of the stimulation protocol, intensity, duration, and interval. Moreover, the efficacy of longer stimulation durations was not tested. Thus, we cannot provide conclusive evidence regarding specific stimulation protocols. All included studies except one were on focal epilepsy patients. Thus, we cannot provide evidence regarding the tDCS effect on different types of epilepsy and other specific syndromes such as Lennox-Gastaut syndrome. Although we performed subgroup analysis due to the presence of heterogeneity, the small number of trials included in each subgroup limits the robustness of our findings. The included studies didn’t provide information on what antiseizure medications were used. Thus, it is not possible to explore the effect of certain medications on the outcome of tDCS. There was a small sample size which limited the representativeness and generalizability of our results. Finally, in this meta-analysis we used the percentage change. Although we are aware of the methodological limitations of such model, it has more relevant and practical interpretation to clinical practice. However, the effect size obtained from it should be interpreted with caution and be supplemented with future larger clinical trials to confirm its robustness.

#### Recommendations

Larger-sample controlled trials with longer follow-up should be carried out. Research should focus on various epilepsy types, including specific epileptic syndromes to understand the scope and limitations of the use of tDCS. Additionally, more studies should be conducted on children to compare the effect of tDCS on different age groups. Other indicators such as the quality of life assessment should be implemented in the outcomes assessed. Also, we recommend putting a consensus primary guideline for the tDCS protocol of application including the site of application, duration of session, number of sessions, and duration of treatment. Moreover, a standardized method of IED assessment should be made to avoid measurement bias. This includes the duration of recording, patient state either awake or asleep, EEG electrode positioning, and the technology used either automated or manual.

## Conclusion

DRE is a major problem that affects patients socially, cognitively, and economically. The tDCS represents a useful adjuvant tool that can help those patients. Among all included studies, seizure reduction percentage ranged from 40 to 84%. This technique showed efficacy in decreasing both seizure frequency and epileptic discharges with tolerable, and self-limiting side effects.

## Electronic supplementary material

Below is the link to the electronic supplementary material.

Supplementary Material 1

## Data Availability

Data will be available from the first or corresponding author upon reasonable request.
