# Supplementary material for: Efficacy of transcranial direct current stimulation on seizure control in patients with refractory epilepsy: a systematic review and meta-analysis of randomized controlled trials
Source: Neurosurg Rev. 2025 Jun 19;48(1):515. doi: 10.1007/s10143-025-03657-0 (PMC12177013; doi:10.1007/s10143-025-03657-0)
Supplement: Supplementary file 1 — Supplementary Material 1 [file 10143_2025_3657_MOESM1_ESM.docx]

**Journal of Neurosurgical Reviews**

**Efficacy of Transcranial Direct Current Stimulation on Seizure Control in Patients with Refractory Epilepsy: A Systematic Review and Meta-analysis of Randomized Controlled Trials.**

*Authors*

Nada Ibrahim Hendi, MBBCh^1^, Yaser AbuSammour, BSc^2^, Ahmed S. Mohamed, BSc^3^, Mohamed Khaled, BSc^4^, Ahmed Mostafa Amin^5^, BSc, Mohamed Saleh Fallaha, BSc^1^, Basma Kamel, BSc^6^, Yehia Nabil Abdalla Helmy, MBBCh^7^, Mostafa Meshref, MSc^8^, Mohammad Ali Saeed Hassan^8,9^

***Affiliations***

^1^ Faculty of Medicine, Ain Shams University, Cairo, Egypt

^2^ Faculty of Medicine, Al-Balqa Applied University, Al-salt, Jordan

^3^ Faculty of medicine, Alexandria university

^4^ Faculty of Medicine Merit University, Sohag, Egypt

^5^ Faculty of Medicine, AL-Azhar University, Cairo, Egypt

^6^ Faculty of Medicine, Mansoura University

^7^ Faculty of Medicine - Zagazig University

^8^ Department of Neurology, Faculty of Medicine, Al-Azhar University, Cairo, Egypt

**Corresponding Author**

Nada Ibrahim Hendi

[nada.i.hendi@gmail.com](mailto:nada.i.hendi@gmail.com)

Table of Contents

[Table S1: Search Strategy	3](#_Toc197987977)

[Table S2: GRADE Assessment	4](#_Toc197987978)

[Table S3: Baseline characteristics of patients in the included trials	5](#_Toc197987979)

[Seizure Frequency Outcome	7](#_Toc197987980)

[Fig. S1: Percentage reduction of seizure frequency based on the time of assessment	8](#_Toc197987981)

[Fig. S2: percentage reduction of seizure frequency based on the number of sessions	9](#_Toc197987982)

[Epileptiform Discharge Outcome	10](#_Toc197987983)

[Fig. S3: Forest plot of the percentage reduction of epileptiform discharge based on the intensity of stimulation	11](#_Toc197987984)

[Fig. S4: Forest plot of the percentage reduction of epileptiform discharge based on the duration of stimulation	12](#_Toc197987985)

[Fig. S5: Forest plot of the percentage reduction of epileptiform discharge based on the number of stimulation sessions	13](#_Toc197987986)

[Meta Regression	14](#_Toc197987987)

[Fig. S6: Meta Regression for seizure frequency outcome	15](#_Toc197987988)

[Fig. S7: Meta Regression for epileptiform discharge outcome	16](#_Toc197987989)

[Cumulative Meta-analysis	17](#_Toc197987990)

[Fig. S8: Cumulative meta-analysis of seizure frequency outcome	18](#_Toc197987991)

[Sensitivity Analysis	20](#_Toc197987992)

[Table S4: Sensitivity analysis	21](#_Toc197987993)

[Table S5: PRISMA Checklist	23](#_Toc197987994)

# Table S1: Search Strategy

("Direct current stimulation" OR tDCS OR "non-invasive stimulation" OR "Trans-cranial electric stimulation" OR "transcranial magnetic stimulation" OR "Trans-cranial magnetic stimulation" OR TMS OR TBS OR neuro-stimulation OR neurostimulation OR "transcranial stimulation" OR "Trans-cranial stimulation" OR "theta burst stimulation" OR "alternating current stimulation" OR tACS OR "Random noise stimulation" OR tRNS OR "trigeminal nerve stimulation")

**AND**

(“Drug resistant” OR drug-resistant OR refractory OR intractable OR pharmacoresistant OR pharmaco-resistant OR uncontrolled OR “treatment resistant” OR treatment-resistant OR medication-resistant OR “medication resistant” OR “Lennox Gastaut” OR “continuous spike and waves”) AND (epilepsy OR seizure OR convulsion OR syndrome)

# Table S2: GRADE Assessment

| **Certainty assessment** | | | | | | | **№ of patients** | | **Effect** | | **Certainty** | **Importance** |
| --- | --- | --- | --- | --- | --- | --- | --- | --- | --- | --- | --- | --- |
| **№ of studies** | **Study design** | **Risk of bias** | **Inconsistency** | **Indirectness** | **Imprecision** | **Other considerations** | **tDCS** | **Sham** | **Relative (95% CI)** | **Absolute (95% CI)** |  |  |
| **Seizure frequency (follow-up: mean 4 weeks)** | | | | | | | | | | | | |
| 10 | randomised trials | serious^a^ | serious^b^ | not serious | serious^c^ | none | 145 | 114 | - | SMD **0.99 SD lower** (1.44 lower to 0.54 lower) | ⨁◯◯◯ Very low | CRITICAL |
| **Epileptiform discharge (follow-up: mean 4 weeks)** | | | | | | | | | | | | |
| 7 | randomised trials | not serious | not serious | not serious | serious^c^ | none | 91 | 60 | - | SMD **1.17 SD lower** (1.67 lower to 0.66 lower) | ⨁⨁⨁◯ Moderate | IMPORTANT |

**CI:** confidence interval; **SMD:** standardised mean difference

#### Explanations

a. Studies included showed a higher risk of bias due to poor allocation concealment, leading to potential randomization bias.

b. Substantial statistical heterogeneity.

c. Due to small sample size.

| Table S3: Baseline characteristics of patients in the included trials | | | | | | | | | | | |
| --- | --- | --- | --- | --- | --- | --- | --- | --- | --- | --- | --- |
| **STUDY ID** | **GROUP NAME** | **AGE** | **Seizure onset age** | **SEX**  **(male no (%))** | **Baseline Seizure frequency mean ± SD** | **IEDS per 30 min** | **Number of antiepileptic drugs n (%)** | | | | |
|  |  |  |  |  |  |  | **1** | **2** | **3** | **4** | **5** |
| **Fregni 2006** | Intervention | 24.3 ± 6.4 | NA | 5 (50%) | 7.2 ± 3.6 (monthly) | 413.9 ± 427.1 | NA | 6(60%) | 4(40%) | NA | NA |
|  | control | 24.0 ± 9.8 | NA | 6 (66%) | 8.4 ± 5.6 (monthly) | 334.4 ± 619.5 | NA | 7(77.7%) | 2(22.2%) | NA | NA |
| **auvichayapat 2013** | Intervention | 11.80 ± 2.1 | 7.3 ± 1.2 | 20 (74%) | 11.67 ± 4.63 (monthly) | 538.44 ± 279.25 | NA | 6(22.2%) | 13(48%) | 5(18.5%) | 3(11.1%) |
|  | control | 11.12 ± 1.6 | 6.8 ± 2.3 | 6 (66%) | 10.22 ± 1.72(monthly) | 582.00 ± 244.19 | NA | 3(33.3%) | 3(33.3%) | 1(11.1%) | 2(22.2%) |
| **auvichayapat 2016** | Intervention | 6.67 ± 1.54 | 2.32 ± 2.39 | 9 (60%) | 80.67 ± 54.43 (daily) | 640.13 ± 263.30 | NA | NA | 9(60%) | 4(26.6%) | 2(13.3%) |
|  | control | 6.29 ± 1.98 | 1.58 ± 1.70 | 5 (71%) | 93.43 ± 59.94(daily) | 800.86 ± 374.62 | NA | NA | 3(42.8%) | 2(28.5%) | 2(28.5%) |
| **Tekturk 2016** | Crossover | 35.42 ± 6.9 | 14.67 ± 8.24 | 6 (50%) | 10.58±9.92 (monthly) | 10.58 ± 9.91 | 1 (8.3%) | 4 (33.3%) | 5 (41.7%) | 2 (16.7%) | NA |
| **Zoghi 2016** | Intervention | 38 ± 13 | NA | NA | 53 ± 78.95(monthly) | NA | NA | NA | NA | NA | NA |
|  | control | 38 ± 13 | NA | NA | 20.28 ± 33.69(monthly) | NA | NA | NA | NA | NA | NA |
| **assenza 2017** | Crossover | 42 ± 15.7 | NA | 4 (40%) | NA | NA | NA | NA | NA | NA | NA |
| **San-Juan 2017** | Intervention 1 | 26–55 | NA | 6 (50%) | 7.167± 3.407 (monthly) | 25.917 ± 41.679 | NA | 5(41.6%) | 5(41.6%) | 2(16.6%) | NA |
|  | Intervention 2 | 27–48 | NA | 4 (50%) | 9.125± 8.919(monthly) | 31.125 ± 74.832 | NA | 4(50%) | 3(37.5%) | 1(12.5%) | NA |
|  | control | 28–65 | NA | 6 (75%) | 11.375± 9.41(monthly) | 13.625 ± 17.287 | NA | 4(50%) | 4(50%) | NA | NA |
| **Yang 2020** | Intervention 1 | 30.5 ± 10.4 | NA | 12 (50%) | 5 ±6.2 (weekly) | NA | 3(12.5%) | 12(50%) | 9(37.5%) | | |
|  | Intervention 2 | 31.8 ± 9.25 | NA | 18 (72%) | 3.9 ±4.5(weekly) | NA | 3(12%) | 11(44%) | 11(44%) | | |
|  | control | 30.19 ± 13 | NA | 12 (57%) | 4 ±7.3(weekly) | NA | 3(14%) | 2(9%) | 10(47.6%) | | |
| **Rezakhani 2022** | Intervention | 26.50 ± 6.5 | 15.80 ± 5.20 | 6(60%) | 8.20 ± 2.39 (monthly) | 10.10 ± 2.38 | NA | NA | NA | NA | NA |
|  | control | 29.30 ± 9.1 | 18.20 ± 5.39 | 5 (50%) | 8.40 ± 2.27(monthly) | 10.00 ± 2.31 | NA | NA | NA | NA | NA |
| **Ashrafzadeh 2023** | Intervention | 9.67 ± 3.08 | NA | 8(88.8%) | 12.89 ± 19.38 (weekly) | 54.22 ± 29.05 | NA | 4(44.4%) | 5(55.5%) | NA | NA |
|  | control | 9.22 ± 3.89 | NA | 6(66.6%) | 16.78 ± 11.3(weekly) | 35.1 ± 17.07 | NA | 1(11.1%) | 8(88.8%) | NA | NA |

# Seizure Frequency Outcome

## Fig. S1: Percentage reduction of seizure frequency based on the time of assessment

## Fig. S2: percentage reduction of seizure frequency based on the number of sessions

# Epileptiform Discharge Outcome

## Fig. S3: Forest plot of the percentage reduction of epileptiform discharge based on the intensity of stimulation

## Fig. S4: Forest plot of the percentage reduction of epileptiform discharge based on the duration of stimulation

## Fig. S5: Forest plot of the percentage reduction of epileptiform discharge based on the number of stimulation sessions

# Meta Regression

## Fig. S6: Meta Regression for seizure frequency outcome

**B)**

A- Meta-regression of seizure frequency based on the age (P value = 0.142), B- Meta-regression of seizure frequency based on the number of sessions (P value = 0.026).

## Fig. S7: Meta Regression for epileptiform discharge outcome

**A)**

**B)**

A- Meta-regression of epileptiform discharge based on the age (P value = 0.796), B- Meta-regression of epileptiform discharge based on the number of sessions (P value = 0.506).

# Cumulative Meta-analysis

## Fig. S8: Cumulative meta-analysis of seizure frequency outcome

Fig. S9: Cumulative meta-analysis of epileptiform discharge outcome

B)

# Sensitivity Analysis

| **A. Results from the sensitivity analyses to Seizure frequency outcome** | | | | |
| --- | --- | --- | --- | --- |
| **Analyses** | **Results** | | | |
| **According to time of assessment** | **SMD (95% confidence interval)** | | **P** | **I^2^** |
| At weeks 1,3 and 8  Serial excluding individual studies at a time | No effect on significance of the effect estimate nor homogeneity | | | |
| At week 2  - Excluding Yang  - Excluding Auvichaypat 2016 | -0.80 (-1.59, -0.02)  -1.85 (-2.36, -1.33) | | 0.04  <0.00001 | 28%  20% |
| At week 4  Removing high risk of bias studies (Ashrafzadeh, Yang) | -0.86 (-1.22, -0.51) | | <0.00001 | 0% |
| **According to Stimulation intensity** | | | | |
| For 1 mA current subgroup  Excluding Fregni | -0.61 (-1.64, 0.42) | 0.68 | | 30% |
| For 2mA current subgroup  - Excluding Auvichaypat 2016  - Excluding Yang | -1.32 (-1.74, -0.90)  -0.84 (-1.26, -0.42) | | <0.00001  <0.0001 | 33%  0% |

# Table S4: Sensitivity analysis

| **According to Number of sessions** | | | | | | |
| --- | --- | --- | --- | --- | --- | --- |
| For 1 session subgroup  Not applicable as limited number of studies | | NA | | | | |
| For >1 session subgroup  - Excluding Yang  - Excluding high risk of bias studies | | -0.66 (-1.11, -0.20)  -0.84 (-1.26, -0.42) | 0.005  <0.0001 | | | 30%  0% |
| **According to Duration of sessions** | | | | | | |
| For 20-min stimulation subgroup  Serial excluding individual studies at a time | | Heterogeneity not resolved. However, it resolves by removing both Fregni and Yang withour affecting the significance. | | | | |
| For 30-min subgroup  - Excluding Tekturk | | -0.72 (-1.20, -0.24) | 0.003 | | | 0% |
| **B. Results from the sensitivity analyses to Epileptiform discharge outcome*** | | | | | | |
| **According to time of assessment** | | | | | | |
| **Immediately after treatment**  **-** Excluding Fregni  - Excluding San-Juan | -0.23(-0.86, 0.40)  -0.88 (-1.39, -0.37) | | | **0.48**  **0.0008** | **50%**  **0%** | |
| **Week 2**  **-** Serial individual study exclusion | **No effect on significance or homogeneity** | | | | | |
| **Week 4**  **-** Excluding Ashrafzadeh  **-** Excluding Fregni | -1.38 (-1.79, -0.98)  -1.04 (-1.54, -0.54) | | | **<0.00001**  **<0.0001** | **0%**  **39%** | |
| **Week 8**  **-** Serial individual study exclusion | **No effect on significance or homogeneity** | | | | | |
| **According to current intensity** | | | | | | |
| **1 mA intensity**  **-** Excluding Ashrafzadeh | -1.74 (-2.43, -1.06) | | | **<0.00001** | **0%** | |
| **2 mA intensity**  **Serial individual study exclusion** | **No effect on significance or homogeneity** | | | | | |
| **According to number of sessions** | | | | | | |
| **Serial individual study exclusion** | **No effect on significance or homogeneity** | | | | | |
| **According to current duration** | | | | | | |
| **20-min stimulation**  **-**Excluding Ashrafzadeh | -1.74 (-2.43, -1.06) | | | **<0.00001** | **0%** | |
| **30-min stimulation**  **Serial individual study exclusion** | **No effect on significance or homogeneity** | | | | | |

* Reporting the studies that had changed significance or heterogenity when excluded

# Table S5: PRISMA Checklist

| Section/topic | # | Checklist item | Reported on page # |
| --- | --- | --- | --- |
| **TITLE** | | | |
| Title | 1 | Identify the report as a systematic review, meta-analysis, or both. | Page 1 |
| **ABSTRACT** | | | |
| Structured summary | 2 | Provide a structured summary including, as applicable: background; objectives; data sources; study eligibility criteria, participants, and interventions; study appraisal and synthesis methods; results; limitations; conclusions and implications of key findings; systematic review registration number. | Page 3 |
| **INTRODUCTION** | | | |
| Rationale | 3 | Describe the rationale for the review in the context of what is already known. | Page 5 |
| Objectives | 4 | Provide an explicit statement of questions being addressed with reference to participants, interventions, comparisons, outcomes, and study design (PICOS). | Page 6 |
| **METHODS** | | | |
| Protocol and registration | 5 | Indicate if a review protocol exists, if and where it can be accessed (e.g., Web address), and, if available, provide registration information including registration number. | Page 7, Paragraph 1 |
| Eligibility criteria | 6 | Specify study characteristics (e.g., PICOS, length of follow-up) and report characteristics (e.g., years considered, language, publication status) used as criteria for eligibility, giving rationale. | Page 7, Paragraph 3 |
| Information sources | 7 | Describe all information sources (e.g., databases with dates of coverage, contact with study authors to identify additional studies) in the search and date last searched. | Page 7, Paragraph 2 |
| Search | 8 | Present full electronic search strategy for at least one database, including any limits used, such that it could be repeated. | eTable 1 in the supplement & page 7 paragraph 2 |
| Study selection | 9 | State the process for selecting studies (i.e., screening, eligibility, included in systematic review, and, if applicable, included in the meta-analysis). | Page 7, Paragraph 3 |
| Data collection process | 10 | Describe method of data extraction from reports (e.g., piloted forms, independently, in duplicate) and any processes for obtaining and confirming data from investigators. | Page 8, Paragraph 1 |
| Data items | 11 | List and define all variables for which data were sought (e.g., PICOS, funding sources) and any assumptions and simplifications made. | Page 7 |
| Risk of bias in individual studies | 12 | Describe methods used for assessing risk of bias of individual studies (including specification of whether this was done at the study or outcome level), and how this information is to be used in any data synthesis. | Page 8, Paragraph 2 |
| Summary measures | 13 | State the principal summary measures (e.g., risk ratio, difference in means). | Page 8, Paragraph 3 |
| Synthesis of results | 14 | Describe the methods of handling data and combining results of studies, if done, including measures of consistency (e.g., I^2^) for each meta-analysis. | Page 8, Paragraph 3 |
| Risk of bias across studies | 15 | Specify any assessment of risk of bias that may affect the cumulative evidence (e.g., publication bias, selective reporting within studies). | Page 9, Paragraph 3 |
| Additional analyses | 16 | Describe methods of additional analyses (e.g., sensitivity or subgroup analyses, meta-regression), if done, indicating which were pre-specified. | Page 9, Paragraph 1 & 2 |
| RESULTS | | | |
| Study selection | 17 | Give numbers of studies screened, assessed for eligibility, and included in the review, with reasons for exclusions at each stage, ideally with a flow diagram. | Page 10, Paragraph 1 |
| Study characteristics | 18 | For each study, present characteristics for which data were extracted (e.g., study size, PICOS, follow-up period) and provide the citations. | Table 1,  eTable 2 in the supplement |
| Risk of bias within studies | 19 | Present data on risk of bias of each study and, if available, any outcome-level assessment (see Item 12). | Page 10, Paragraph 3 |
| Results of individual studies | 20 | For all outcomes considered (benefits or harms), present, for each study: (a) simple summary data for each intervention group and (b) effect estimates and confidence intervals, ideally with a forest plot. | Page 11-13 |
| Synthesis of results | 21 | Present results of each meta-analysis done, including confidence intervals and measures of consistency. | Page 11-13 |
| Risk of bias across studies | 22 | Present results of any assessment of risk of bias across studies (see Item 15). | Page 10, Paragraph 3 |
| Additional analysis | 23 | Give results of additional analyses, if done (e.g., sensitivity or subgroup analyses, meta-regression [see Item 16]). | Page 13, Paragraph 4 & page 14, paragraph 1,2 |
| DISCUSSION | | | |
| Summary of evidence | 24 | Summarize the main findings including the strength of evidence for each main outcome; consider their relevance to key groups (e.g., health care providers, users, and policy makers). | Page 15, paragraph 1 |
| Limitations | 25 | Discuss limitations at study and outcome level (e.g., risk of bias), and at review level (e.g., incomplete retrieval of identified research, reporting bias). | Page 17, Paragraph 2 |
| Conclusions | 26 | Provide a general interpretation of the results in the context of other evidence, and implications for future research. | Page 18, Paragraph 2 |
| FUNDING | | | |
| Funding | 27 | Describe sources of funding for the systematic review and other support (e.g., supply of data); role of funders for the systematic review. | Page 18, Paragraph 4 |
